# Unique Case of Dislocation of Both Lenses into the Anterior Chamber

**Published:** 1881-10

**Authors:** A. W. Calhoun

**Affiliations:** Atlanta, Ga.; Professor of Diseases of the Eye, Ear, and Throat in the Atlanta Medical College


### UNIQUE CASE OF DISLOCATION OF BOTH LENSES INTO THE ANTERIOR CHAMBER.

By A. W. CALHOUN, M.D., Atlanta, Ga.
Professor of Diseases of the Eye, Ear, and Throat in the Atlanta Medical College.

Mr. E. C. H., of Alabama, aged thirty years, a stout, healthy man, extremely near-sighted since early life, happened to an unusual accident, which finally resulted in great good to his formerly very defective vision.

While superintending the hauling of some farm produce, occasion offered for testing his great strength, which he demonstrated by lifting one end of a heavily loaded four-horse wagon, out of a rut. He exerted himself to the very utmost, straining almost every muscle in his body. mmediately upon coming out from underneath the wagon, the right eye gave him much pain, vision became obscured, the outlines of objects appearing indistinct and distorted. Violent inflammation with intense suffering followed, confining him to his bed for five weeks, during which time he was under the treatment of his attending physicians. As soon as practicable, he sought special treatment, and upon examination, I found the following state of things:

The conjunctiva was very much inflamed and swollen (œdema), and both photophobia and lachrymation existed to a painful degree. The pupil was widely dilated, only a narrow rim of the iris being apparent. In the anterior chamber, resting against the posterior surface of the cornea, was the dislocated lens, now partially cataractous, and moving to and fro in the aqueous humor, reminding one very forcibly of the movements of a very large oil globule in water. The vision was practically gone, by reason of the beginning opacity of the lens. I recognized the condition of the eye at once, and after hearing the history of the case, was satisfied the dislocation took place at the moment of his making the violent effort to lift the wagon, the strain having ruptured the suspensory ligament and forced the lens through the pupil into the anterior chamber. He was chloroformed, an opening made at the lower corneo-sclerotic junction (as in cateract extraction by the lower flap operation) and the lens removed.

The recovery was rapid, and in a short time he was going about, using of course the uninjured eye. As before mentioned, he had previously been near-sighted to such a degree, as only to be able to recognize objects quite near him, even with the aid of *concave* glasses. Now by the aid of proper *convex* glasses, the vision is brought up to $\frac{20}{30}$ and both near and distant objects are clear and distinct. In other words, a near-sighted eye, with defective vision is transformed by a serious accident, into a far-sighted eye with almost normal vision.

But the most uncommon part of the case is yet to come. In returning home, he jumped upon a rapidly moving train, and fell upon the floor of the car with considerable force. The same peculiar sensations began instantly in the previously good (left) eye, that occurred in the right after the strain from lifting. He returned to my office the following day, when I found the transparent dislocated lens of the left eye floating about in the anterior chamber, resembling more closely than in the other eye, a drop of oil in the aqueous humor. The same operation was made as before, with the same good result. This eye had been more near-sighted than the other and the vision more defective, but the adjustment of a convex glass increased it to $\frac{20}{50}$; thus converting it also, in the absence of the lens, into a far-sighted eye, with greatly increased sight.

The dislocation of the left lens was due immediately to the fall in the cars, but no doubt the straining which caused the complete rupture of the suspensory ligament of the right lens, also partially ruptured the ligament of the left lens and the fall completed it. Fortunately the dislocated lenses passed through the readily yielding pupils into the anterior chambers, otherwise they would have fallen into the vitreous humor and done irreparable damage by their presence.

It is now five years since the last operation. A few days ago, the patient presented himself for the first time since then, "simply to inform me, his eyes were still good."

The pupils remained permanently and widely dilated in this case, and so far as my observation and experience goes, this permanent dilatation after such injuries is the rule. The abnormal pressure of the lens upon the iris, paralyzes its muscular tissue, and no amount of treatment will cause these muscular fibres to contract and thus bring the pupil down to its natural size. No doubt the injury producing the dislocation, is itself in many instances, in part, the cause of the iritic paralysis and dilatation of the pupil.

Another point of great interest, is the fact that myopic or near-sighted balls are more subject to dislocation of the lens, than any others. This no doubt is due to the weakening of the suspensory ligament of the lens, through the diseased elongation of the ball, which takes place in all cases of myopia. At all events, most of the cases of this character, which have come into my hands, have been in near-sighted persons, and this can hardly be said to be simply a coincidence, when we know that "a near-sighted eye is a diseased eye," and peculiarly subject to very serious evils in the interior of the ball. The abnormal shape of a near-sighted ball and the abnormal action of the ocular muscles of such an eye, with the tendency to disease on the interior, make dislocation of the lens an easy possibility in the myope.

Dislocation of the lens is common enough, but that one lens should be displaced by an accident into the anterior chamber, and after a short interval the same thing happen to the other eye, and both eyes be brought into better visual condition than ever by means of an operation, is a rarity which induces this report.

